# Integrated Mapping of Neglected Tropical Diseases: Epidemiological Findings and Control Implications for Northern Bahr-el-Ghazal State, Southern Sudan

**DOI:** 10.1371/journal.pntd.0000537

**Published:** 2009-10-27

**Authors:** Hugh J. W. Sturrock, Diana Picon, Anthony Sabasio, David Oguttu, Emily Robinson, Mounir Lado, John Rumunu, Simon Brooker, Jan H. Kolaczinski

**Affiliations:** 1 Malaria Consortium, Juba, Southern Sudan; 2 London School of Hygiene and Tropical Medicine, London, United Kingdom; 3 Vector Control Division, Ministry of Health, Government of Uganda, Kampala, Uganda; 4 Malaria Consortium – Africa Regional Office, Kampala, Uganda; 5 Ministry of Health, Government of Southern Sudan, Juba, Southern Sudan; 6 Kenya Medical Research Institute (KEMRI)-Wellcome Trust Research Programme, Nairobi, Kenya; Centers for Disease Control and Prevention, United States of America

## Abstract

**Background:**

There are few detailed data on the geographic distribution of most neglected tropical diseases (NTDs) in post-conflict Southern Sudan. To guide intervention by the recently established national programme for integrated NTD control, we conducted an integrated prevalence survey for schistosomiasis, soil-transmitted helminth (STH) infection, lymphatic filariasis (LF), and loiasis in Northern Bahr-el-Ghazal State. Our aim was to establish which communities require mass drug administration (MDA) with preventive chemotherapy (PCT), rather than to provide precise estimates of infection prevalence.

**Methods and Findings:**

The integrated survey design used anecdotal reports of LF and proximity to water bodies (for schistosomiasis) to guide selection of survey sites. In total, 86 communities were surveyed for schistosomiasis and STH; 43 of these were also surveyed for LF and loiasis. From these, 4834 urine samples were tested for blood in urine using Hemastix reagent strips, 4438 stool samples were analyzed using the Kato-Katz technique, and 5254 blood samples were tested for circulating *Wuchereria bancrofti* antigen using immunochromatographic card tests (ICT). 4461 individuals were interviewed regarding a history of ‘eye worm’ (a proxy measure for loiasis) and 31 village chiefs were interviewed regarding the presence of clinical manifestations of LF in their community. At the village level, prevalence of *Schistosoma haematobium* and *S. mansoni* ranged from 0 to 65.6% and from 0 to 9.3%, respectively. The main STH species was hookworm, ranging from 0 to 70% by village. Infection with LF and loiasis was extremely rare, with only four individuals testing positive or reporting symptoms, respectively. Questionnaire data on clinical signs of LF did not provide a reliable indication of endemicity. MDA intervention thresholds recommended by the World Health Organization were only exceeded for urinary schistosomiasis and hookworm in a few, yet distinct, communities.

**Conclusion:**

This was the first attempt to use an integrated survey design for this group of infections and to generate detailed results to guide their control over a large area of Southern Sudan. The approach proved practical, but could be further simplified to reduce field work and costs. The results show that only a few areas need to be targeted with MDA of PCT, thus confirming the importance of detailed mapping for cost-effective control.

## Author Summary

Integrated control of neglected tropical diseases (NTDs) is being scaled up in a number of developing countries, because it is thought to be more cost-effective than stand-alone control programmes. Under this approach, treatments for onchocerciasis, lymphatic filariasis (LF), schistosomiasis, soil-transmitted helminth (STH) infection, and trachoma are administered through the same delivery structure and at about the same time. A pre-requisite for implementation of integrated NTD control is information on where the targeted diseases are endemic and to what extent they overlap. This information is generated through surveys that can be labour-intensive and expensive. In Southern Sudan, all of the above diseases except onchocerciasis require further mapping before a comprehensive integrated NTD control programme can be implemented. To determine where treatment for which disease is required, integrated surveys were conducted for schistosomiasis, STH infection, LF, and loiasis, throughout one of ten states of the country. Our results show that treatment is only required for urinary schistosomiasis and STH in a few, yet separate, geographical area. This illustrates the importance of investing in disease mapping to minimize overall programme costs by being able to target interventions. Integration of survey methodologies for the above disease was practical and efficient, and minimized the effort required to collect these data.

## Introduction

Southern Sudan is one of a few African countries that have recently embarked on integrated control of some of their endemic neglected tropical diseases (NTDs) [1]. Integration typically targets those diseases for which safe and effective preventive chemotherapy (PCT) is readily available and often made possible through drug donation programmes. The main NTDs endemic in Southern Sudan for which mass drug administration (MDA) of PCT forms a key part of control initiatives are onchocerciasis, lymphatic filariasis (LF) caused by *Wuchereria bancrofti* infection, soil-transmitted helminths (STH: hookworms, *Ascaris lumbricoides* and *Trichuris trichiura*), schistosomiasis (due to *Schistosoma haematobium* and *S. mansoni*), and trachoma [2]. It is anticipated that combining MDA of PCT in areas where these diseases overlap will allow control of NTDs that are presently not being targeted and improve cost-effectiveness compared to stand-alone control programmes [3].

Appropriate targeting of integrated MDA requires information on the geographical distribution of different NTDs, to identify areas that would benefit most from this approach [4]. In the absence of such information, priority areas for control or elimination and the estimation of drug requirements is often based on expert opinion or out-of-date information. Absence of reliable evidence can result in delivery of drugs in areas where different NTDs are rare or absent, or in more frequent MDA than required for effective control, thus wasting drugs and resources as well as exerting unnecessary selection pressure on parasites, which may lead to development of drug resistance.

In Southern Sudan, up until now, the distribution of onchocerciasis has been comprehensively mapped by the Ministry of Health, Government of Southern Sudan (MoH-GoSS) with assistance from the African Program for Onchocerciasis Control [5]. Trachoma surveys have also been undertaken in a number of areas in the country [6]–[8]. For LF, schistosomiasis and STH, by contrast, systematic prevalence data are not available, with previous studies in Southern Sudan having been few and limited in scale [9]–[11]. In addition, historic data indicate that *Loa loa* is endemic in the southern states (referred to as the Equatoria region) [12], which has been confirmed through RAPLOA assessments in onchocerciasis endemic areas. However, the extent of this parasite beyond the known endemic state of Western Equatoria, as well as in LF endemic areas outside the onchocerciasis foci in the Equatoria region, has not been clearly delineated [5]. In the absence of detailed information on *L. loa*, ivermectin coverage for LF elimination cannot be safely expanded beyond onchocerciasis endemic areas, because of the risk of severe and sometimes fatal adverse events to ivermectin treatment in individuals with high levels of *L. loa* infection. For these reasons, it was deemed necessary to conduct comprehensive surveys for schistosomiasis, STH infection, LF, and loiasis across the country to guide the nationwide design and implementation of an integrated PCT package.

Currently, the World Health Organization (WHO) recommends that the MDA need for LF elimination is determined through lot quality assurance sampling (LQAS) of 250 individuals in each intervention unit (typically a district or equivalent administrative unit) [13]. LQAS surveys should be preceded by a review of existing information on LF and rapid assessments through questionnaires, seeking information on the prevalence of clinical manifestations of *W. bancrofti* infection (hydrocele and lymphoedema) from key informants [13]. Based on questionnaire data it should then be possible to demarcate areas as endemic, non-endemic or as being of undetermined endemicity. For schistosomiasis and STH control, WHO recommends that 200–250 school-aged children are sampled in each ecological zone [14]. Assessments for loiasis should be conducted in communities earmarked for inclusion in MDA campaigns of ivermectin and be based on interviews of 80 adults per community regarding a history of eye worm [15]. At present, however, experience on how best to integrate surveys for schistosomiasis, STH infection, LF and loiasis is limited, as is guidance on what constitutes an integrated survey.

A pilot project in Nigeria, aiming to incorporate LF elimination and urinary schistosomiasis control into an existing onchocerciasis control programme, undertook rapid surveys of LF and urinary schistosomiasis in 149 villages in two states [16]. In the survey, LF assessments were conducted among 30 male volunteers using the immunochromatographic card test [ICT] and *Schistosoma haematobium* prevalence was determined using reagent strips among 30 randomly selected school children during school surveys conducted in the same villages. The small sample size adopted in the survey meant that areas with low infection prevalence, especially of LF, might have been missed. Thus, there remains a need for further operational experience on integrated surveys for LF and schistosomiasis, but also for STH and loiasis. Here we report on results of an integrated NTD survey in Northern Bahr-el-Ghazal, the first state in Southern Sudan to integrate the delivery of up to three anthelmintic drugs to treat multiple NTDs as part of nationwide scaling-up.

The survey focused on urinary and intestinal schistosomiasis, STH infection, and LF. Information was also collected on reported loiasis symptoms, using the recommended rapid assessment procedure [15]. Information was not collected on the prevalence of trachoma, since trachoma surveys require a different set of skills and sampling frame [17]. The overall purpose of the integrated survey was to generate data by which it could be decided whether MDA of PCT is required for a specific intervention unit (in this case the payam or areas within this administrative area), rather than to generate precise prevalence estimates for a given geographical area. We also mapped the results of the survey, to identify any spatial patterns in infection, and to highlight focal problems. The implications of our findings for delivery of integrated NTD control are discussed, as are the possibilities for modifying the survey protocol, based on our practical experience.

## Methods

### Ethical considerations

The study protocol received ethical approval from the Directorate of Research, Planning and Health System Development, MoH-GoSS, and from the Ethics Committee of the London School of Hygiene and Tropical Medicine, UK. Clearance to conduct the surveys was obtained from the State MoH, followed by County Health Departments. The study was explained to each member of the selected households. The household heads were asked to provide written consent for the entire household to participate in the study. The study was then explained to each household inhabitant who met the inclusion criteria, and s/he was asked to consent verbally to participating in the study; only those who provided consent were registered and requested to provide samples. Due to the large number of individuals that were surveyed, it was considered impractical to obtain written consent from each study participant, and the institutional review boards approved this procedure. To document verbal consent, the name of each individual who provided verbal consent was recorded, along with the test results for their samples. Individuals that tested positive for schistosomiasis or STH infection were treated with praziquantel or albendazole respectively, according to WHO guidelines. The four individuals positive for *W. bancrofti* antigen were not treated on site, but informed of their infection and its possible implications, after which they were referred for treatment to the nearest health facility. On the basis of advice from the MoH-GoSS, this approach was considered more appropriate, as repeated treatment will be required to kill microfilaria and thus prevent transmission.

### Study site

The survey was conducted in 86 villages in Northern Bahr-el-Ghazal State, north-western Southern Sudan during February to May 2009. In Southern Sudan, the first administrative unit is the state, followed by county (2^nd^) and payam (3^rd^). Northern Bahr-el-Ghazal State is divided into five counties and 22 payams, and has an estimated population of 1,580,695, which amounts to about 12% of the total population of Southern Sudan. The State experiences a single rainy season, typically between June and September. The population mainly consists of the Dinka ethnic group, which engages in nomadic cattle herding at riverside camps during the dry season and growing of millet and other varieties of grain in fixed settlements during the rainy season. Like most of Southern Sudan, this State is characterized by a lack of physical infrastructure and occasional insecurity, making the conduct of surveys particularly challenging.

### Sample population and selection

The survey was based on an integrated NTD survey protocol developed by the MoH-GoSS and Malaria Consortium [18], with financial support from the US Agency for International Development (USAID) and technical input from the Centers for Disease Control and Prevention and RTI International. This protocol followed WHO recommendations for each of the NTDs, with slight modifications to improve feasibility in the challenging context of Southern Sudan. In each payam, a two-stage, quasi-random sampling approach was employed. Initially, in order to maximise identification of high LF prevalence areas, selection of villages was based on anecdotal reports on the presence of lymphoedema and hydrocele, collected through interviews with payam administrative and medical staff. In accordance with WHO recommendations, sampling for LF in each payam was conducted until a maximum of 250 individuals had tested negative, which required visits to up to three villages. In addition, in those villages selected for LF surveys, we interviewed village chiefs regarding the presence of clinical manifestations of LF in their village. The majority of villages surveyed for LF were also surveyed for schistosomiasis and STH. Due to delays with the supply of ICT kits, 13 villages that should have been surveyed for both LF and schistosomiasis/STH were instead only selected based on proximity to water and only surveyed for schistosomiasis/STH. When ICT kits were available, 13 different villages were selected for LF surveys, based on anecdotal reports of clinical manifestations. Individuals were excluded from the study if they had not lived in the payam for at least six months or did not provide informed consent.

For schistosomiasis and STH, the number of villages to be sampled in each payam was calculated according to the population size of each payam, whilst ensuring a minimum of two geographically well-separated sites were selected per payam. To guide selection of villages in addition to those already selected through our LF sampling strategy, a list of villages within areas of expected schistosomiasis risk was generated. This list was derived from an initial map of expected risk based on climatic and ecological information. However, due to the lack of a georeferenced village database, a list of villages close to water bodies was compiled during interviews with payam administrative staff and a random selection was taken. If this selection procedure did not generate a sufficient number of villages, additional ones were chosen by using a randomly generated list.

STH infection was assumed to be geographically more homogeneously distributed than schistosomiasis and LF [19]–[21], and therefore selection of sites on the basis of LF and schistosomiasis ecology was considered sufficient to capture the inherent spatial heterogeneity of STH infection. Such sampling approach was based on the operational requirement to identify whether a particular payam required MDA of PCT for a particular NTD, rather than to statistically estimate the prevalence of species infection.

Coordinates for each village were collected using handheld GPS devices (eTrex, Garmin International, Kansas, U.S.A.).

### Survey methods

In each selected village, meetings were held with village elders to explain the nature and purpose of the survey. In those villages where LF surveys were conducted, we also interviewed the village chief regarding residents with clinical manifestations of LF, using the standard WHO questionnaire [13]. Initially, households were selected using the random walk approach [22]; however, in some villages households were too dispersed for this method to be feasible, and registration of individuals subsequently occurred at a central location. For schistosomiasis and STH, children aged 5 to 15 years were invited to participate, and households were selected until a sample of 70 children was registered in each village. Selected children were given containers for stool and urine samples, and asked to drop the sample off at a central point, where the field laboratory had been established.

For LF, individuals aged 16 years and above were invited to participate, and households were selected until a total of 110 adults had been registered in each village. Those registered were requested to provide a finger-prick blood sample to be tested for circulating *W. bancrofti* antigen using an immunochromatographic card tests (ICT) (BinaxNOW Filariasis, Inverness Medical). ICT kits were refrigerated whilst in storage in Nairobi, Juba and Aweil, according to guidelines, and were kept in cool boxes throughout fieldwork. In accordance with WHO guidelines [13], if one or more tests had been positive in a sample of 100 individuals, then no further testing would have been undertaken. In practice however, this scenario did not occur in any payam, and therefore a second site in the payam had to be selected, with a further 110 adults registered and requested to provide a finger-prick blood sample. In addition, those children registered for schistosomiasis and STH in the selected village were also requested to provide a finger-prick blood sample so that a total of 180 individuals were registered for LF at the second site. If there were insufficient individuals in the second village to reach a total sample size of 250, then sampling continued in a neighbouring, third, village. In villages surveyed for LF, data on the presence of *L. loa* were collected from each adult registered for ICT testing using the WHO recommended RAPLOA rapid assessment procedure [15]. This procedure consists of asking adults three questions regarding the presence of worms in their eyes. Consistent with WHO recommendations, children tested for LF were not interviewed for *L. loa*.

Parasitological examination of stool and urine samples was conducted in the field by a team of trained laboratory technicians. Faecal samples were examined in duplicate for *S. mansoni* and STH ova using the Kato-Katz method within an hour of preparation, to avoid the clearing of hookworm eggs. Urine samples were tested for haematuria using Hemastix reagent strips (Bayer Corporation), with test positive urine samples subsequently examined using urine filtration [23]. Most technicians were skilled in conducting all of the survey tasks and were regularly rotated between: registration of individuals for the survey, preparation of stool and urine slides for microscopic examination, collecting finger prick blood samples for ICT kits and carrying out microscopic examination of slides.

### Data analysis

The data were double-entered into Microsoft Excel. Data checks and first entry were conducted at the end of each survey day. Second entry was mostly conducted during the period of field work and completed afterwards. Range and consistency checks were conducted for all variables. Maps of infection prevalence were developed using ArcGIS 9.2 (ESRI, California, U.S.A.) and ecological correlates of infection were investigated. Environmental variables included in the analysis were mean normalized difference vegetation index (NDVI) [24] and distance to nearest perennial water bodies (http://www.fao.org/geonetwork/srv/en/main.home). Maximum Land Surface Temperature was not included in either model as temperatures only ranged from 53.5–55.6°C, which was thought to be not a biologically relevant range over which to model. Binomial logistic regression models were initially developed to identify significant predictors of infection using STATA 10.0 (Stata Corporation, College Station, TX, U.S.A.) and covariates with Wald's P>0.05 were included, together with age and sex, into a spatially explicit Bayesian logistic regression model. The model took into account the clustered nature of the data, using WinBUGS version 1.4 (MRC Biostatistics Unit, Cambridge, and Imperial College London, UK), following methodology detailed by Clements et al. [25].

## Results

In total, 4904 children and 4834 adults from 86 villages across Northern Bahr-el-Ghazal State were registered to take part in the survey. Only Agoga payam, in the north of the state, was inaccessible by vehicle during the study period. For schistosomiasis and STH infection, 73 villages were surveyed and a total of 4450 stool samples and 4597 urine samples were examined (Dataset S1). For LF, 5254 blood samples from 43 villages were tested. Two of the sites surveyed for LF had insufficient inhabitants to make up the required sample, thus necessitating inclusion of individuals from a neighbouring village. These data were merged before analysis due to the close proximity of the sites.

Of the children that provided either a stool or urine sample, the mean age was 8.9 years (inter-quartile range (IQR): 7–11 years) and 50% were male. Of the adults that provided a blood sample, the mean age was 36.7 years (IQR: 25–45 years) and 36.6% were male.

**Figure 1:**
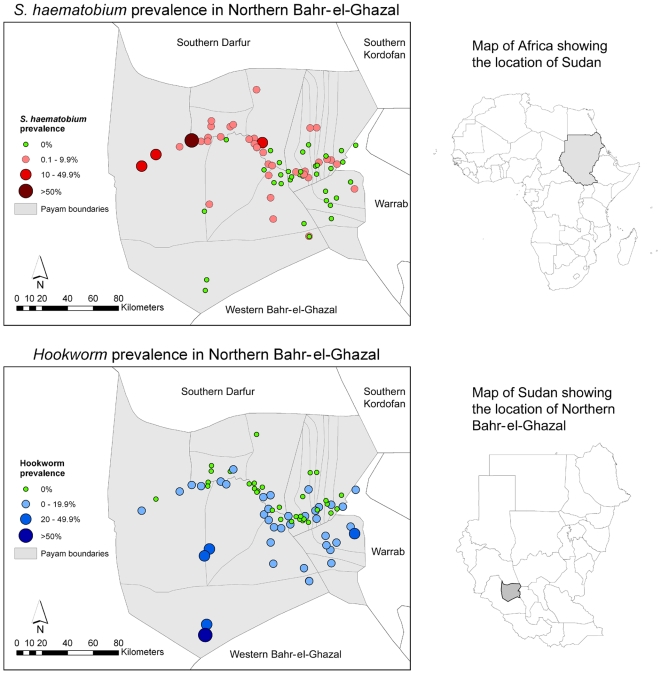
The distribution of *S. haematobium* and hookworm in Northern Bahr-el-Ghazal State.

The overall prevalence of *S. haematobium* was 3.0% (0–65.6% by village) and *S. mansoni* was 0.2% (0–4.2% by village) (Figure 1, Dataset S1). Although state-wide levels of infection were low, there was marked geographical variation, with prevalence of *S. haematobium* >20% in some villages along the Loll river. The most common STH infection found in the State was hookworm: the overall prevalence was 4.9% (0–70% by village) (Figure 1, Dataset S1). Hookworm prevalence showed a strong geographical pattern, exceeding 20% in the south of the State. *A. lumbricoides* was only detected in one individual and no individuals were found to be positive for *T. trichiura*. The Bayesian logistic regression showed no significant associations between environmental factors and infection patterns (results not shown), but that children aged 10–15 years were 2.46 times more likely (95% Bayesian Credible Interval: 1.54, 3.94) to be infected with *S. haematobium* than children aged 5–10 years.

For LF, no payams had antigenaemia prevalence above 1%; overall only four ICT positives were detected, two each in Aweil Centre and Aweil East payam (Dataset S1). Questionnaires on the presence of clinical manifestations of LF in the community were administered to village chiefs in 31 of the 43 villages surveyed with ICTs. In 84% (26) and 71% (22) of villages, respondents reported having seen villagers with elephantiasis or hydrocele, respectively. Questionnaire data were only available for two of the four villages with positive ICT results, in both of which respondents reported having seen one case of elephantiasis and no or ten cases of hydrocele. All other villages with reported cases of elephantiasis or hydrocele were ICT negative. History of eye worm, calculated according to WHO guidelines [15], was reported in only four individuals from two villages.

In terms of treatment, WHO guidelines recommend that in communities with schistosomiasis prevalence of >10% and <50%, school-aged children and high risk groups of adults should be treated with praziquantel once every two years. In communities where prevalence is ≥50%, the same groups should be treated once a year [2]. In Northern Bahr-el-Ghazal State, *S. haematobium* infection exceeded these MDA thresholds in only four of the survey communities, one of which qualifies for annual MDA (Dataset S1). Pooling the village prevalence data at the payam level would mean that one payam, Ayat, could be categorized as ‘eligible’ for MDA, because overall prevalence of *S. haematobium* infection was 20.2%.

For STH, WHO recommends delivery of MDA with either albendazole or mebendazole once a year to pre-school and school-aged children, as well as to pregnant women and high risk groups of adults, where cumulative prevalence of STH is >20% and <50%, and twice a year where prevalence is >50% [2]. In Northern Bahr-el-Ghazal State only five of the survey communities exceeded the MDA threshold for STH (Dataset S1), due to the presence of hookworm, with only one community falling into the biannual MDA category. Pooling of survey data at the payam level would result in two payams, Aroyo and Awoda, exceeding the MDA intervention threshold, with a mean hookworm prevalence of 26.9% and 56.5%, respectively. Of the payams that did not qualify for MDA only one had a study village with a prevalence of *S. haematobium* of >10% and none of the payams had a cumulative STH prevalence of >20%.

## Discussion

A sound understanding of NTD distribution and prevalence is an essential prerequisite for cost-effective control, with each national programme needing to be tailored to its specific context. MDA should be targeted to those areas and populations in greatest need and programme managers hence require information on populations at risk and numbers to be treated, to estimate the funding needed to deliver the intervention. Here we present results from the first integrated survey for schistosomiasis, STH infection, LF, and loiasis in Southern Sudan, which was conducted to generate information on the distribution of infection to help guide the scaling up of integrated NTD control.

Results showed that older children were more at risk of being infected with *S. haematobium* than children between ages five to ten years, consistent with previous studies in the region [26],[27]. The lack of association between *S. haematobium* and environmental factors contrasts studies in Tanzania and West Africa that show an association with distance to water bodies [28],[29]. The observed lack of association in Northern Bahr-el-Ghazal may be due to very high temperatures, limiting the number of water bodies with suitable temperatures for snail survival. Equally, the fact that a large proportion of sites included in the schistosomiasis survey were selected based on their proximity to water bodies may have masked a statistical association between infection and environmental risk factors.

Overall prevalence of schistosomiasis and hookworm was low, but varied markedly between villages. The highest prevalence of *S. haematobium* occurred along the Loll river, which runs from West to East through the State. *S. haematobium* has previously been reported in Southern Darfur [30], but this is, we believe, the first survey to be conducted in Northern Bahr-el-Ghazal State. The snail intermediate host *Bulinus truncatus* is generally found in Southern Darfur where it is well adapted to pools and slow-flowing waters and seems to be able to tolerate drought [31]. The occurrence of *S. mansoni* in southern Darfur – and potentially in Northern Bahr-el-Ghazal State – may be due to population movements from the Gezira irrigation area in central Sudan [32]. This absence of hookworm in northern areas of the State is consistent with studies on the distribution of STH infection in neighbouring Chad [32], as is the absence of *A. lumbricoides* and *T. trichiura* with other studies in Southern Sudan [11], presumably due to thermal exclusion [33].

The finding that *W. bancrofti* prevalence was consistently below the WHO recommended intervention threshold of 1% for LF elimination is somewhat surprising, given that all states in northern Sudan have been classified as LF endemic [34] and that recent ICT-based surveys in Upper Nile State and in the Equatoria region of Southern Sudan all reported antigenaemia prevalences exceeding the 1% threshold [5]. Clinical data collected by WHO Juba through questionnaires sent to health care providers in Warrap State, adjacent to Northern Bahr-el-Ghazal State, as well as in Lakes and Jonglei States, also indicated that these areas have been exposed to LF transmission, as hydrocele and lymphoedema were not uncommon [5]. This existing evidence for LF transmission led us to expect that LF extended into Northern Bahr-el-Ghazal State, an assumption that is not supported by our findings. Given the large number of sites sampled and their wide geographical distribution, we consider it unlikely that a focus of LF infection would have been missed. Instead, it may be that the distance and climatic difference between confirmed LF foci in the region and the current survey area were too large to have allowed the establishment of sustained transmission. Although we do not know the location of the sites surveyed in northern Sudan, it is clear that the nearest confirmed focus in Southern Sudan (Tambura County, Western Equatoria) is at least 150 km from the border of the State surveyed here. This distance exceeds the estimated average 50 km diameter of LF foci that provides the basis for suggested RAGFIL rapid assessment procedures [35]. Based on this estimate, the Tambura focus would not be expected to extend to Northern Bahr-el-Ghazal unless the area in-between (Western Bahr-el-Ghazal State) was favourable for LF transmission. To date there have been no reports of LF infection or clinical manifestations from this State; confirmative surveys will be required to determine how far north the Tambura focus extends. The small number of individuals positive for LF in Northern-Bahr-el-Ghazal could potentially be due to historic infections of individuals elsewhere who subsequently migrated to the state. We were unable to verify this assertion, as potential study participants were only asked whether they had been resident in the area for at least six months to meet inclusion criteria. We recommend that future surveys collect additional detail on residency from study participants.

We found no obvious correlation between reported (by village chiefs and recorded on standard WHO forms [13]) clinical manifestations of LF and ICT results. The majority of respondents reported having seen local inhabitants with hydrocele or elephantiasis, in some villages more than 10 cases of either manifestation, but only in two of these did we find ICT positive individuals. These results are consistent with findings from Nigeria where patients were asked if they had a swollen scrotum and subsequently examined for hydrocele and tested with an ICT [36]. Asking about scrotal swelling was not predictive for presence of hydrocele, and calculating hydrocele prevalence at the community level only reliably indicated whether a village was ICT positive if hydrocele prevalence exceeded 20%. Asking village chiefs in Southern Sudan about the presence of elephantiasis or hydrocele among local residents is likely to suffer from similar shortcomings, due to underreporting (village chiefs will not be aware of all/most cases) and due to the fact that individuals with clinical manifestations are as likely as not to have an active infection [37]. There is a need therefore for further evaluation of the use of chronic conditions caused by LF as a proxy for active infection in Southern Sudan.

In addition to understanding possible explanations for observed results, it is also important to recognize the limitations of the survey, due, in part, to the difficult context of operating in Southern Sudan. First, the lack of up-to-date census data and a georeferenced village database – due to longstanding civil war – meant that villages could not be selected entirely at random from within areas identified to be at risk of schistosomiasis. Instead, local knowledge had to be used to identify sites where schistosomiasis (as well as clinical manifestations of LF) had been reported from and that were accessible. Second, the use of purposive sampling may have resulted in slightly higher prevalence estimates when data were pooled at the payam level; this however is unlikely to have substantially affected the classification of payams. Third, in the more dispersed villages it was not feasible to implement a random walk selection procedure and a convenience sample was selected at a central point. This approach may have introduced sampling bias through (i) individuals with potential clinical signs of disease being more likely to attend because of the offer of diagnosis and treatment and (ii) ill individuals unable to attend [38]. However, village leaders were used to mobilise individuals, and although bias may have been introduced, it is unlikely to have altered the overall treatment classifications. Recent experience of a filariasis treatment coverage survey in Haiti found little difference between coverage estimates obtained through a convenience sample of houses near distribution points and a cluster survey [39]. Fourth, a number of those individuals that tested positive for schistosomiasis or hookworm reported having migrated to the area within the last two years. As the lifespan of both adult *S. haematobium* and hookworms is between 3–7 years [40],[41], the inclusion of individuals that recently settled in the area may have over-estimated active transmission in some villages. Fifth, the age group surveyed for STH and schistosomiasis included children between the ages of 5–15 years, whereas children between the ages of 10–15 years are normally targeted because prevalence typically peaks in this age group. The decision to use a wider age band was made to maximise the number of children surveyed in a village so as to avoid having to visit a second village. During preliminary visits we had found that villages are often small and would not contain sufficient 10–15 year olds to complete sampling. Given the results from the statistical analysis, it is likely that our prevalence estimate for schistosomiasis is slightly lower than would have been the case if only 10–15 year old children had been included. However, prevalence in individuals aged 5–9 is still expected to be high for both STH and schistosomiasis [42], and any differences by age groups are unlikely to have affected the classification of villages in the current study. There was no statistical difference in prevalence between these two age classes for hookworm infection. Despite the effect of these limitations on the accuracy of our prevalence estimates, we believe that they are unlikely to have had a major effect on the study results and that the implications for targeting of NTD control in Northern Bahr-el-Ghazal State remain valid.

The results clearly show that MDA for LF is not required, while some areas should be targeted with albendazole or praziquantel for treatment of hookworm or urinary schistosomiasis, respectively. How these areas are comprised, i.e. whether they should constitute the entire payam or only parts of it, should follow a pragmatic approach. We propose the following priority areas: 1) The whole eligible population of those payams with a number of hookworm endemic sites and where, consequently, mean prevalence exceed the WHO recommended intervention threshold of 20% (i.e. Aroyo and Awoda payams), should be targeted with an annual round of albendazole. This is justified because albendazole is relatively cheap at USD 0.02 per treatment, and STH infections are less focal than is the case with schistosomiasis; 2) Starting in the payam that has a number of moderately to highly endemic *S. haematobium* villages (i.e. Ayat payam in the West of the State), communities within 5 km of the Loll river should receive praziquantel biennially. Resource permitting this strategy should be extended along the river to the east. This more geographically targeted approach should be applied because of the focal distribution of schistosomiasis, its clear association with water bodies [20],[30], and because an adult dose of praziquantel costs between USD 0.20–0.30 and is prone to cause more side-effects than albendazole, particularly in populations that have not been previously treated [2]. These proposed treatment campaigns should be conducted during the remaining months of the current dry season. In future, finer targeting of schistosomiasis control should be considered to conserve scarce resources. For urinary schistosomiasis, WHO recommends the use of blood in urine questionnaire, ideally distributed through the existing school system. However, there are currently very few schools in Southern Sudan and although the questionnaire may be distributed through community networks, it would still require validation before this method is put to routine use [4].

Integrated surveys, like integrated control of NTDs, are intuitively appealing as this approach has the potential to minimize field work and associated costs. However, the implementation of different sampling designs by the same survey team does not necessarily imply an integrated survey. There are a number of inherent differences in the recommended mapping approach for each NTD that need to be considered and reconciled [4], including: (i) the target age group for sampling; (ii) the number of individuals to be sampled, which partly depends on the prevalence threshold used to denote the need for MDA; (iii) the inherent variation in the spatial heterogeneity of infection between the different NTDs; and (iv) the various tests and approaches used to diagnose infection (urine, stool and blood). In the present survey, we adopted a balanced, pragmatic approach to sampling both villages and individuals within selected villages. For example, once LF villages had been selected, we selected further villages based on the potential risk of schistosomiasis. Also, where insufficient adults were identified for LF sampling, we included children who had been selected for stool and urine examination. In an integrated survey of trachoma and malaria in Ethiopia, the same individuals from selected households underwent eye examination and provided blood samples for malaria parasite detection [43]. Although not formally assessed, the authors of the Ethiopian study concluded that the integration of two surveys was not only done without significant increases in the time required in the field or effort of the field teams, but only incurred the extra cost of one fieldworker. Similarly, it is expected that the integrated survey design described here may have yielded cost savings over stand-alone surveys for each of the diseases. At present, however, there is little evidence for such cost saving, or indeed on the feasibility and cost-effectiveness of integrated survey designs. This is a topic of current investigation (Sturrock et al, unpublished data; Kolaczinski et al, unpublished data).

Given the results of the current surveys, further mapping is clearly required to determine MDA needs in the other nine states of Southern Sudan. This provides the opportunity use the experience gained during this first survey to modify the protocol, aiming to further enhance efficiency. To do so, one option would be to reduce the number of survey sites. Based on the observed relative homogeneity of STH in the study area and elsewhere [19], future surveys may include fewer villages than in the present study to reach decisions regarding treatment strategies. Future investigation of this possibility is warranted and needs to be based on an empirical understanding of the (i) inherent spatial heterogeneity of STH infection in different settings; (ii) financial and human costs of conducting epidemiological surveys; and (iii) consequences for control of misclassifying an administrative unit; this is an area of ongoing research (Hugh Sturrock, unpublished data). Similarly, for schistosomiasis it may be feasible to reduce the number of sites initially sampled to one per payam and use reagent sticks and circulating cathodic antigen tests for detection of blood in urine for *S. haematobium* and *S. mansoni*, respectively [4]. As in the current study, results from individuals found to be positive when tested with rapid diagnostic tests could be confirmed by microscopy. Also, when a site is found to be positive for schistosomiasis, further surveys could be carried out in the payam to gain a better understanding of disease distribution. Such surveys could possibly be carried out using a rapid sampling approach such as LQAS. This technique has been shown to be practical and more cost-effective than treating an entire administrative area (such as payam), due to the highly spatially heterogeneous nature of schistosomiasis [20],[44]. Finally, we recommend that the use of questionnaires for LF and urinary schistosomiasis assessments should be further evaluated in Southern Sudan, to assess the sensitivity and specificity of this method over a range of transmission settings.

### Conclusion

These results represent the most up to date epidemiological data on schistosomiasis, STH infection and LF collected in Southern Sudan. In addition, we report the first integrated survey design for this group of parasitic infections. The survey results show that within Northern Bahr-el-Ghazal State, the only NTDs of public health importance are: i) *S. haematobium*, where the area along the Loll River qualifies for biennial MDA with praziquantel, and ii) hookworm, where two payams are affected and should be targeted with annual albendazole or mebendazole. The findings from these surveys highlight that anecdotal data and reports from health care providers or key informants are insufficient to determine treatment needs. To develop a national endemicity maps for the target NTDs to direct resources for integrated control, the remaining states of Southern Sudan will need to be surveyed for presence of infection. Based on the experience from this first round of surveys, we have discussed ways in which such surveys could be conducted more rapidly and cheaply, while generating results of equal reliability.

## Supporting Information

Dataset S1 Village-level prevalence data from integrated NTD survey sites in Northern Bahr-el-Ghazal State, Southern Sudan
(0.24 MB DOC)

Checklist S1 STROBE checklist
(0.08 MB DOC)
