# Supplementary material for: Integrated Mapping of Neglected Tropical Diseases: Epidemiological Findings and Control Implications for Northern Bahr-el-Ghazal State, Southern Sudan
Source: PLoS Negl Trop Dis. 2009 Oct 27;3(10):e537. doi: 10.1371/journal.pntd.0000537 (PMC2761732; doi:10.1371/journal.pntd.0000537)
Supplement: Dataset S1 — Village-level prevalence data from integrated NTD survey sites in Northern Bahr-el-Ghazal State, Southern Sudan (0.24 MB DOC) [file pntd.0000537.s001.doc]

Table. Village level summary of the results of integrated NTD survey in Northern Bahr-el-Ghazal. (a) Represents survey sites selected according to the system outlined in the methods. (b) represents sites where, due to problems with ICT kits, LF was surveyed alone.

|  | **County** | **Payam** | **Village** | **Urine samples** | ***S. haematobium* prevalence (%)** | **Stool samples** | **Hookworm prevalence (%)** | ***S. mansoni prevalence (%)*** | **Blood samples** | **ICT positive (%)** | **Loa Loa prevalence (%)** |
| --- | --- | --- | --- | --- | --- | --- | --- | --- | --- | --- | --- |
| **(a)** | Aweil | Aroyo | Aroyo Town | 73 | 1.4 | 65 | 30.8 | 0 | NA | - | - |
|  | Centre | Aroyo | Nykuala/Mattar | 57 | 0 | 54 | 22.2 | 9.3 | 152 | 0 | 0 |
|  |  | Aweil Town | Hai Salaam | 51 | 3.9 | 49 | 6.1 | 0 | 135 | 0 | 0 |
|  |  | Awoda | Manbiel | 48 | 0 | 45 | 35.6 | 0 | NA | - | - |
|  |  | Awoda | Kanwanji | 71 | 0 | 70 | 70 | 0 | NA | - | - |
|  |  | Barmayen | War ager | 76 | 0 | 74 | 17.6 | 0 | 148 | 0 | 1.84 |
|  |  | Barmayen | Burayak | 60 | 0 | 60 | 10 | 0 | 99 | 0 | 0 |
|  | Aweil | Baac | Mathiandit | 65 | 4.6 | 68 | 0 | 0 | NA | - | - |
|  | East | Baac | Paraick | 77 | 5.2 | 72 | 1.4 | 0 | NA | - | - |
|  |  | Baac | Mayanadot | 72 | 0 | 63 | 1.6 | 0 | NA | - | - |
|  |  | Baac | Baac | 63 | 3.2 | 59 | 0 | 1.7 | 99 | 0 | 0 |
|  |  | Baac | Wiyon / Oun | 67 | 0 | 68 | 1.5 | 0 | NA | - | - |
|  |  | Baac | Burrap | 49 | 0 | 46 | 0 | 0 | NA | - | - |
|  |  | Madhol | Mabong Tong | 67 | 1.5 | 61 | 11.5 | 0 | NA | - | - |
|  |  | Madhol | Dhaik | 61 | 0 | 61 | 0 | 0 | NA | - | - |
|  |  | Madhol | Panamei | 71 | 1.4 | 73 | 0 | 0 | NA | - | - |
|  |  | Madhol | Rumbol | 60 | 0 | 60 | 0 | 0 | NA | - | - |
|  |  | Malualbai | Ajakriek | 57 | 0 | 61 | 0 | 0 | NA | - | - |
|  |  | Malualbai | Peth | 72 | 0 | 62 | 3.2 | 1.6 | NA | - | - |
|  |  | Malualbai | Adoor | 62 | 1.6 | 63 | 0 | 0 | NA | - | - |
|  |  | Malualbai | Amath | 65 | 0 | 65 | 1.5 | 0 | NA | - | - |
|  |  | Mangartong | Burakuoc | 70 | 1.4 | 70 | 0 | 0 | NA | - | - |
|  |  | Mangartong | Njangmir | 71 | 0 | 71 | 1.4 | 0 | NA | - | - |
|  |  | Mangok | Wuluch | 59 | 0 | 57 | 1.8 | 0 | 96 | 0 | 0 |
|  |  | Mangok | Wat Malual/Ayach | 17 | 0 | 18 | 0 | 0 | 49 | 0 | 2.04 |
|  |  | Mangok | Lietnhom/Warawar diluit | 67 | 0 | 65 | 3.1 | 0 | 104 | 0.96 | 0 |
|  |  | Wunlang | Ador Agot | 69 | 1.4 | 64 | 3.1 | 0 | 100 | 0 | 0 |
|  |  | Wunlang | Abyei | 63 | 0 | 62 | 0 | 0 | NA | - | - |
|  |  | Wunlang | Tongoi | 71 | 0 | 69 | 0 | 0 | NA | - | - |
|  |  | Wunlang | Karagala | 16 | 6.3 | 16 | 0 | 0 | NA | - | - |
|  |  | Wunlang | Rum Aihol | 68 | 0 | 72 | 0 | 0 | NA | - | - |
|  |  | Yargot | Halibul | 66 | 4.5 | 61 | 0 | 0 | NA | - | - |
|  |  | Yargot | Marchar Tuop | 64 | 1.6 | 63 | 4.8 | 0 | 100 | 0 | 0 |
|  |  | Yargot | Karmarial | 67 | 0 | 68 | 0 | 0 | NA | - | - |
|  | Aweil | Ariath | Ariath | 65 | 10.8 | 63 | 0 | 0 | 100 | 0 | 0 |
|  | North | Ariath | Amel | 64 | 0 | 60 | 0 | 0 | 162 | 0 | 0 |
|  |  | Ariath | Pandit | 64 | 3.1 | 63 | 0 | 0 | NA | - | - |
|  |  | Malual East | Mayem Ulem | 60 | 0 | 63 | 1.6 | 0 | 100 | 0 | - |
|  |  | Malual East | Mariel Agep | 64 | 0 | 62 | 0 | 0 | NA | - | - |
|  |  | Malual East | Watok | 69 | 1.4 | 61 | 3.3 | 0 | NA | - | - |
|  |  | Malual East | Jaac | 62 | 6.5 | 61 | 0 | 0 | 159 | 0 | 0 |
|  |  | Malual West | Pamat | 63 | 3.2 | 58 | 0 | 0 | 101 | 0 | 0 |
|  |  | Malual West | Gok Machar | 62 | 8.1 | 57 | 0 | 0 | 161 | 0 | 0 |
|  |  | Malual West | Tit Chok | 68 | 2.9 | 61 | 0 | 0 | NA | - | - |
|  |  | Malual West | Makuach Piol | 64 | 3.1 | 62 | 0 | 0 | NA | - | - |
|  |  | Malual West | Majak Kar | 67 | 1.5 | 65 | 1.5 | 0 | NA | - | - |
|  |  | Malual West | Mabok Kuot | 61 | 1.6 | 61 | 6.6 | 0 | NA | - | - |
|  | Aweil | Gakrol | Acen Mac | 57 | 0 | 57 | 3.5 | 0 | 100 | 0 | 0 |
|  | South | Gakrol | Akac | 61 | 0 | 62 | 11.3 | 0 | 150 | 0 | 0 |
|  |  | Mangargir | Panthau | 51 | 0 | 51 | 13.7 | 0 | 100 | 0 | 0 |
|  |  | Mangargir | Pankar | 65 | 0 | 67 | 9 | 0 | 150 | 0 | 0 |
|  |  | Mangargir | Thieralieth | 65 | 1.5 | 61 | 23 | 0 | NA | - | - |
|  |  | Wathmuok | Achuan | 57 | 0 | 53 | 3.8 | 0 | 100 | 0 | 0 |
|  |  | Wathmuok | Rup | 63 | 0 | 64 | 10.9 | 0 | 151 | 0 | 0 |
|  | Aweil | Ayat | Agoor | 65 | 1.5 | 64 | 1.6 | 0 | 100 | 0 | 0 |
|  | West | Ayat | Goren I | 67 | 31.3 | 60 | 6.7 | 1.7 | 159 | 0 | 0 |
|  |  | Ayat | Langic | 62 | 3.2 | 61 | 0 | 0 | NA | - | - |
|  |  | Ayat | Yaal | 64 | 9.4 | 60 | 1.7 | 0 | NA | - | - |
|  |  | Ayat | Makuom | 61 | 65.6 | 57 | 5.3 | 0 | NA | - | - |
|  |  | Ayat | Akuak Rual | 68 | 11.8 | 66 | 0 | 0 | NA | - | - |
|  |  | Gomjuer | Gongrual | 67 | 1.5 | 62 | 0 | 0 | 112 | 0 | 0 |
|  |  | Gomjuer | Makuei | 60 | 5 | 63 | 0 | 0 | NA | - | - |
|  |  | Gomjuer | Nyamlel (Akwangap) | 68 | 0 | 63 | 3.2 | 3.2 | NA | - | - |
|  |  | Gomjuer | Panyieth | 69 | 1.4 | 64 | 0 | 0 | 148 | 0 | - |
|  |  | Gomjuer | Amatnyang | 64 | 4.7 | 62 | 0 | 0 | NA | - | - |
|  |  | Mariem East | Maduany | 69 | 0 | 68 | 2.9 | 0 | 100 | 0 | - |
|  |  | Mariem East | Akong | 67 | 0 | 61 | 1.6 | 0 | NA | - | - |
|  |  | Mariem East | Ngol Mal | 70 | 0 | 63 | 0 | 0 | 148 | 0 | 0 |
|  |  | Mariem West | Luethabur | 67 | 0 | 65 | 4.6 | 0 | 100 | 0 | 0 |
|  |  | Mariem West | Majak Piol | 63 | 1.6 | 61 | 1.6 | 0 | 171 | 0 | 0 |
|  |  | Mariem West | Kolroth | 68 | 1.5 | 65 | 4.6 | 0 | NA | - | - |
|  |  | Mariem West | Mariem | 69 | 1.4 | 59 | 0 | 0 | NA | - | - |
|  |  | Mariem East | Nyalath | 45 | 2.2 | 47 | 2.1 | 0 | NA | - | - |
|  |  |  |  |  |  |  |  |  |  |  |  |
|  |  |  |  |  |  |  |  |  |  |  |  |
| **(b)** | Aweil | Aroyo | Kurchok | NA | - | NA | - | - | 103 | 0 | 0 |
|  | Centre | Aweil Town | Zira | NA | - | NA | - | - | 100 | 1 | 0 |
|  |  | Awoda | Awoda Centre | NA | - | NA | - | - | 114 | 0 | 0 |
|  |  | Awoda | Mantiel/Awoda Centre | NA | - | NA | - | - | 104 | 0.96 | 0 |
|  | Aweil | Baac | War war | NA | - | NA | - | - | 149 | 0 | 0 |
|  | East | Madhol | Madhol | NA | - | NA | - | - | 109 | 0 | 0 |
|  |  | Madhol | Mabong Geng | NA | - | NA | - | - | 152 | 0 | 0 |
|  |  | Malualbai | Malualbai Centre | NA | - | NA | - | - | 106 | 0 | 0 |
|  |  | Malualbai | Manyel | NA | - | NA | - | - | 143 | 0 | 0 |
|  |  | Mangartong | Adhathic | NA | - | NA | - | - | 98 | 0 | 0 |
|  |  | Mangartong | Mangartong Centre | NA | - | NA | - | - | 125 | 0 | 0 |
|  |  | Wunlang | Kergal | NA | - | NA | - | - | 145 | 0 | 0 |
|  |  | Yargot | Atuekchk | NA | - | NA | - | - | 152 | 0.66 | 0 |
|  |  |  |  |  |  |  |  |  |  |  |  |
|  | **Northern Bahr El Gazal** | | | **4597** | **3.0** | **4438** | **4.9** | **0.2** | **5254** | **0.1** | **0.1** |
